# Comparative Transcriptomic Analyses Propose the Molecular Regulatory Mechanisms Underlying 1,8-Cineole from *Cinnamomum kanehirae* Hay and Promote the Asexual Sporulation of *Antrodia cinnamomea* in Submerged Fermentation

**DOI:** 10.3390/molecules28227511

**Published:** 2023-11-09

**Authors:** Huaxiang Li, Jianing Dai, Juanjuan Wang, Chunlei Lu, Zhishan Luo, Xiangfeng Zheng, Zhenming Lu, Zhenquan Yang

**Affiliations:** 1 School of Food Science and Engineering, Yangzhou University, Yangzhou 225009, China; lihuaxiangyu@126.com (H.L.); 13404251799@163.com (J.D.); wangjuanjuanzj@163.com (J.W.); luchunlei7y@163.com (C.L.); zxf@yzu.edu.cn (X.Z.); 2 Ministry of Education Key Laboratory of Industrial Biotechnology, School of Biotechnology, Jiangnan University, Wuxi 214122, China; zhishan_luo33@163.com; 3 National Engineering Research Center of Cereal Fermentation and Food Biomanufacturing, Jiangnan University, Wuxi 214122, China; zhenming_lu@163.com

**Keywords:** *Antrodia cinnamome*, *Cinnamomum kanehirae* Hay, submerged fermentation, asexual sporulation, transcriptomics, 1,8-cineole, molecular regulatory mechanism

## Abstract

*Antrodia cinnamomea* is a valuable edible and medicinal mushroom with antitumor, hepatoprotective, and antiviral effects that play a role in intestinal flora regulation. Spore-inoculation submerged fermentation has become the most efficient and well-known artificial culture process for *A. cinnamomea*. In this study, a specific low-molecular compound named 1,8-cineole (cineole) from *Cinnamomum kanehirae* Hay was first reported to have remarkably promoted the asexual sporulation of *A. cinnamomea* in submerged fermentation (AcSmF). Then, RNA sequencing, real-time quantitative PCR, and a literature review were performed to predict the molecular regulatory mechanisms underlying the cineole-promoted sporulation of AcSmF. The available evidence supports the hypothesis that after receiving the signal of cineole through cell receptors Wsc1 and Mid2, Pkc1 promoted the expression levels of *rlm1* and *wetA* and facilitated their transfer to the cell wall integrity (CWI) signal pathway, and *wetA* in turn promoted the sporulation of AcSmF. Moreover, cineole changed the membrane functional state of the *A. cinnamomea* cell and thus activated the heat stress response by the CWI pathway. Then, heat shock protein 90 and its chaperone Cdc37 promoted the expression of *stuA* and *brlA,* thus promoting sporulation of AcSmF. In addition, cineole promoted the expression of *areA*, *flbA*, and *flbD* through the transcription factor NCP1 and inhibited the expression of *pkaA* through the ammonium permease of MEP, finally promoting the sporulation of AcSmF. This study may improve the efficiency of the inoculum (spores) preparation of AcSmF and thereby enhance the production benefits of *A. cinnamomea*.

## 1. Introduction

*Antrodia cinnamomea* (syn. *Antrodia camphorate* or *Taiwanofungus camphoratus*) is a rare and valuable edible and medicinal fungus that belongs to the phylum Basidiomycetes and family Polyporaceae [1,2]. *A*. *cinnamomea* performs various types of bioactivities, such as hepatoprotective, antitumor, anti-inflammatory, antiviral, vasodilation-inhibiting, hypoglycemic, and immunoregulation [2,3,4]. Four main artificial culture processes for *A. cinnamomea* have been used: wood culture, plate culture, solid-state fermentation, and submerged fermentation (SmF). Compared with traditional mycelial inoculation, inoculation with asexual spores (i.e., arthrospores) produced as inocula through liquid cultivation has the advantages of allowing the control of inoculum size and seed quality; having excellent batch stability, a short fermentation period, and a high yield of bioactive compounds; and being easy to scale up. Thus, spore-inoculation SmF has become the most efficient and popular artificial cultivation method for *A*. *cinnamomea*.

However, the SmF of *A*. *cinnamomea* still has some disadvantages, and the most prominent is the low yield of asexual spores (used as inocula), which results in tedious and time-consuming inoculum preparation and high production costs. Therefore, promoting the sporulation of *A. cinnamomea* in submerged fermentation (AcSmF) is crucial to the efficiency and development of industrial *A*. *cinnamomea* production. Screening compounds that promote the sporulation of AcSmF and underlying molecular mechanisms have been extensively explored [5].

*Cinnamomum kanehirae* Hay is the sole natural host of *A*. *cinnamomea*. It usually grows in high-altitude areas (450–2000 m) with a warm and humid climate and fertile soil [6]. It exerts antibacterial, antiseptic, and anticancer effects [7,8], and many active compounds have been isolated from its leaves and stems, including coumarin, isoscopoletin, scopoletin, ferulic acid, methyl-(21*R*)-pheophoride A, isoobtusilactone A, obtusilactone A, methyl-pheophorbide B, stigmasta-4,22-dien-3-one, *p*-hydroxybenzaldehyde, β-sitostenone, β-sitosterol, 2-methylpropyl benzoate, (+)-yangambin, (+)-syringaresinol, (+)-sesamin, (+)-diasesamin, (+)-episesamin, and 5,4′-dihydroxy-7-methoxyflavone [8,9,10].

Zhang et al. [11] found that the homogenate of *C*. *kanehirae* stems increased the triterpenoid content in the mycelium of *A*. *cinnamomea* during plate cultivation. Comparative transcriptomics analysis has shown that a *C*. *kanehirae* homogenate promotes triterpene synthesis by up-regulating the expression levels of hydroxymethylglutaryl CoA reductase, farnesyl transferase, and squalene synthase genes, which are crucial to triterpene biosynthesis. Zeng et al. [12] found that the ethanol extract of *C*. *kanehirae* leaves effectively promotes mycelial growth and phenol biosynthesis in *A*. *cinnamomea* during solid-state fermentation. Hsu et al. [13] found that polysaccharides from the aqueous extracts of *Cinnamomum camphora* stem can promote mycelial growth in *A*. *cinnamomea*. Lu et al. [14] found that a 0.05 g/L petroleum ether extract of *C. camphora* stem can markedly promote mycelial growth and triterpene biosynthesis in *A*. *cinnamomea*. These studies have demonstrated that some compounds in *C*. *kanehirae* or other *Cinnamomum* plants promote the growth and reproduction of *A*. *cinnamomea,* or active compound biosynthesis.

In this study, four organic solvents (methanol, ethyl acetate, petroleum ether, and chloroform) were used in extracting compounds from *C*. *kanehirae*. Then, the effects of the extracts on the sporulation of AcSmF were compared. The chloroform extract of *C*. *kanehirae* (CkCE) remarkably promoted the sporulation of *A*. *cinnamomea*. Liquid chromatography-mass spectrometry (LC-MS/MS) analysis was performed to detect the composition of CkCE. The main component of CkCE was 1,8-cineole (cineole), which promoted the sporulation of *A*. *cinnamomea*. However, the molecular regulatory mechanism underlying cineole-promoted asexual sporulation has not been uncovered. Therefore, comparative transcriptomics technology using RNA sequencing (RNA-seq) and real-time quantitative PCR (RT-qPCR) was used to predict the molecular regulatory mechanism underlying the cineole-promoted sporulation of AcSmF.

## 2. Results and Discussion

### 2.1. Effect of C. kanehirae Extracts on the Sporulation of AcSmF

**Figure 1:**
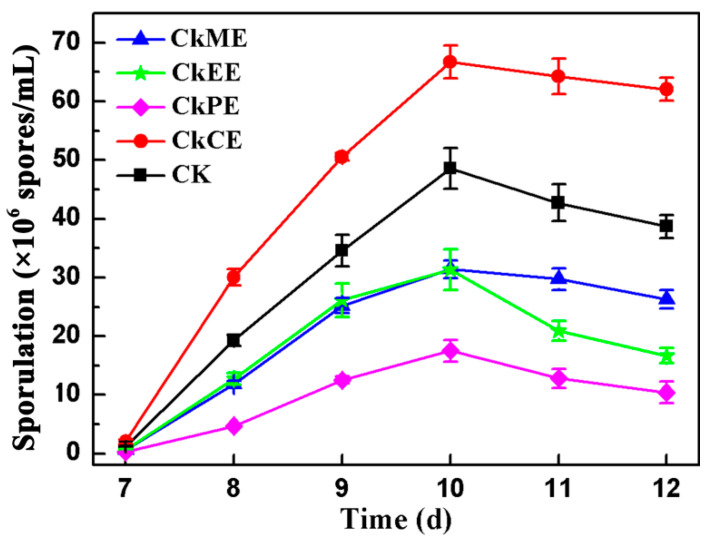
Effects of different extracts of *C. kanehirae* on the sporulation of AcSmF. CK: Control check; CkCE: Chloroform extract of *C. kanehirae*; CkME: Methanol extract of *C. kanehirae*; CkEE: Ethyl acetate extract of *C. kanehirae*; CkPE: Petroleum ether extract of *C. kanehirae*. All extracts were dissolved in dimethyl sulfoxide (DMSO) and added at a concentration of 50 μg/mL. The blank control group was given the same volumes of DMSO.

The effects of 50 μg/mL CkME, CkEE, CkCE, and CkPE on the sporulation of AcSmF were compared (Figure 1). Only CkCE promoted the sporulation of *A*. *cinnamomea* and increased the sporulation by 37.34% compared with the control. The other three extracts inhibited the sporulation of AcSmF. Especially, CkPE decreased the rate of sporulation by 64.01% compared with the control. The results indicated that *C. kanehirae* contains some compounds that promote the sporulation of *A*. *cinnamomea*. The compounds were mainly present in CkCE.

### 2.2. Composition of CkCE

The compounds that promote the sporulation of AcSmF were confirmed by identifying the composition of CkCE through LC–MS/MS. More than 600 substances in CkCE were identified (Supplementary Table S1), mainly including flavonoids, phenols, steroids, coumarins, carboxylic acids, prenol lipids, organonitrogen compounds, and benzene and substituted derivatives. Table 1 lists the top 20 compounds in CkCE. The content of cineole was the highest (20.49%), considerably higher than that of the other compounds. The compounds with a content exceeding 8% were L-histidinol, deoxyuridine, and catechol. However, catechol is an unsuitable additive due to its acute toxicity and carcinogenicity [15], but whether cineole, L-histidinol, and deoxyuridine promote the sporulation of *A*. *cinnamomea* should be further verified.

### 2.3. Effects of the Main Compounds in CkCE on the Sporulation and Biomass of AcSmF

**Figure 2:**
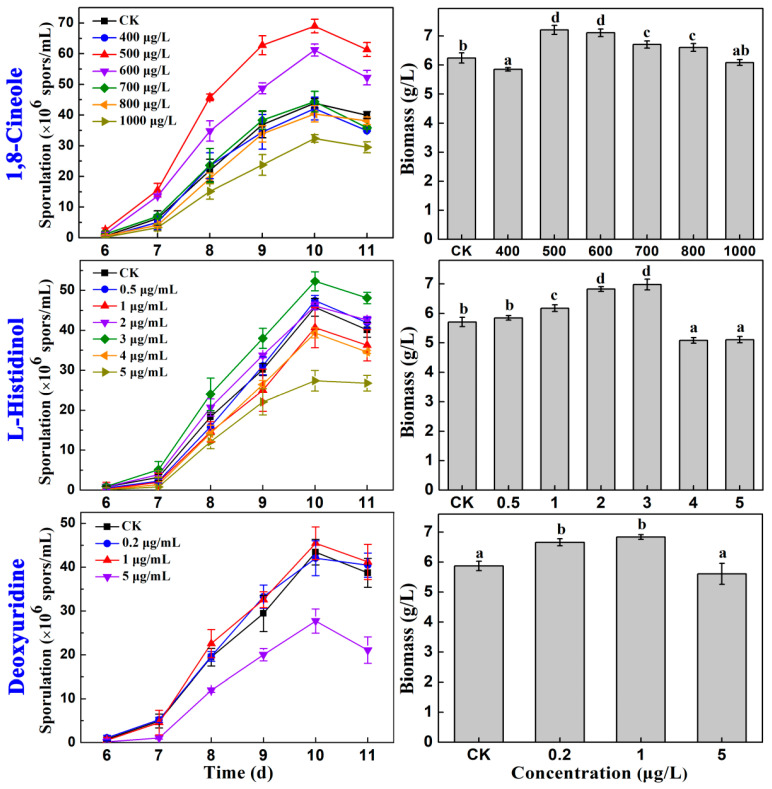
Effects of different concentrations of 1,8-cineole, L-histidinol, and deoxyuridine on the sporulation and biomass of *A*. *cinnamomea* in submerged fermentation. Different letters (a–d) on the column indicate the significant difference at the level of 0.05.

The effects of different concentrations of cineole, L-histidinol, and deoxyuridine on the sporulation and biomass of AcSmF were compared (Figure 2). Deoxyuridine had no obvious effect on the sporulation of *A*. *cinnamomea* but dramatically inhibited the process when its concentration reached 2 μg/mL.

Cineole at concentrations of 500 and 600 μg/L markedly promoted the sporulation and mycelial growth of AcSmF, but 500 μg/L cineole presented a better effect. Cineole at a 500 μg/L concentration increased sporulation (6.90 × 10^7^ spores/mL) by 57.25% relative to that of the control. The effect of cineole was considerably better than that of CkCE (37.74%; Figure 1). In addition, 500 μg/L of cineole increased the biomass by 15.54% relative to that of the control. Sporulation had a larger increase than biomass (57.25% vs. 15.54%). Therefore, cineole promoted the sporulation of *A*. *cinnamomea* by increasing the sporulation capacities of the mycelia rather than increasing the amount of mycelia.

Similarly, 3 μg/mL L-histidinol increased the sporulation of *A*. *cinnamomea* by 14.20% compared with that of the control. However, it increased the biomass of *A*. *cinnamomea* by 22.24% compared with that of the control. Sporulation had a smaller increase than the biomass (14.20% vs. 22.24%). Therefore, L-histidinol promoted the sporulation of *A*. *cinnamomea* by increasing the amount of mycelia rather than by promoting the sporulation of the mycelia.

In summary, cineole was the most important compound in CkCE and promoted the sporulation of *A*. *cinnamomea*. Therefore, the molecular regulatory mechanism underlying the cineole-promoted sporulation of AcSmF was further investigated.

### 2.4. RNA-Seq and Statistical Analysis

#### 2.4.1. Preparation of Samples for RNA-Seq

In the spore-inoculation SmF of *A*. *cinnamomea*, the arthrospores produced by liquid fermentation were first inoculated into the medium. After that, the arthrospores begin to germinate and gradually become longer. After about 32 h, the spore germination rate is close to 100%; after about 48 h, the germinated arthrospores form flocculent hyphae. After fermentation for 3 days, the hyphae begin to entangle with each other; after 5 days, the hyphae become entangled into small balls (mycelial pellets) of uniform size. The shape of the mycelial pellets will no longer change until the end of fermentation. After 6 days of fermentation, the hyphae on the surface of the pellets began to break and form arthrospores. Then, the number of arthrospores keeps increasing until it reaches its maximum at 10 days.

**Figure 3:**
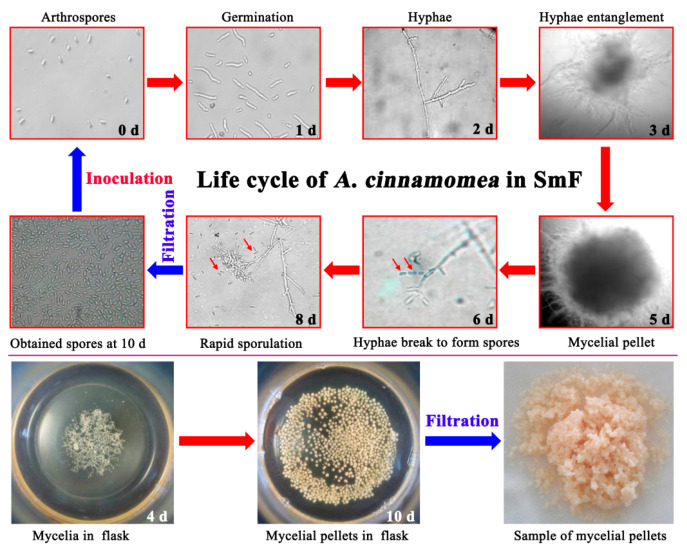
The life cycle pictures (400×) and photographs of *A*. *cinnamomea* in submerged fermentation. All the pictures in the life cycle were taken with an optical microscope (Nikon TE2000S, Tokyo, Japan) at 400 times magnification; the pictures of flasks and mycelial pellets were taken with a cell phone.

Subsequently, abundant arthrospores can be obtained by filtering with gauze, and these arthrospores can be used as inoculum for the next batch of fermentation. Simultaneously, the mycelial pellets were also obtained through filtration. After washing, the arthrospores may adhere to the surface of the pellets, and the mycelia (pellets) can be used for samples for genomic sequencing, RNA sequencing, proteome identification, and metabolome identification. The life cycle and pictures of *A*. *cinnamomea* in SmF are shown in Figure 3. All the life cycle only involves asexual reproduction without any sexual reproduction.

*A*. *cinnamomea* mycelial pellets cultured with 500 μg/L of cineole (marked as “An”) and without cineole (marked as “CK”) were collected and used for RNA-seq. The sporulation curves of *A*. *cinnamomea* with fermentation time (Supplementary Figure S1) indicated the following: (1) the effect of 500 μg/L cineole promoted the sporulation of AcSmF; (2) a few *A*. *cinnamomea* spores were produced on the sixth day, indicating that the sixth day was the initial stage of sporulation; (3) the sporulation rate increased sharply after 6 days and reached a maximum on the eighth day, indicating that the eighth day was the middle stage of sporulation; (4) the sporulation reached a maximum and then began to decline rapidly after 10 days of fermentation, indicating that the 10th day was the late stage of sporulation. Therefore, samples were collected after 6 days of *A*. *cinnamomea* mycelium fermentation (initial stage of sporulation), 8 days (middle stage of sporulation), and 10 days (late stage of sporulation) in the presence of cineole (marked as “An_6d”, “An_8d”, and “An_10d”) and absence of cineole (marked as “CK_6d”, “CK_8d”, and “CK_10d”). Each sample had three replicates and was used for RNA-seq.

#### 2.4.2. Statistical Analysis of Sample Repeatability and DEGs

**Figure 4:**
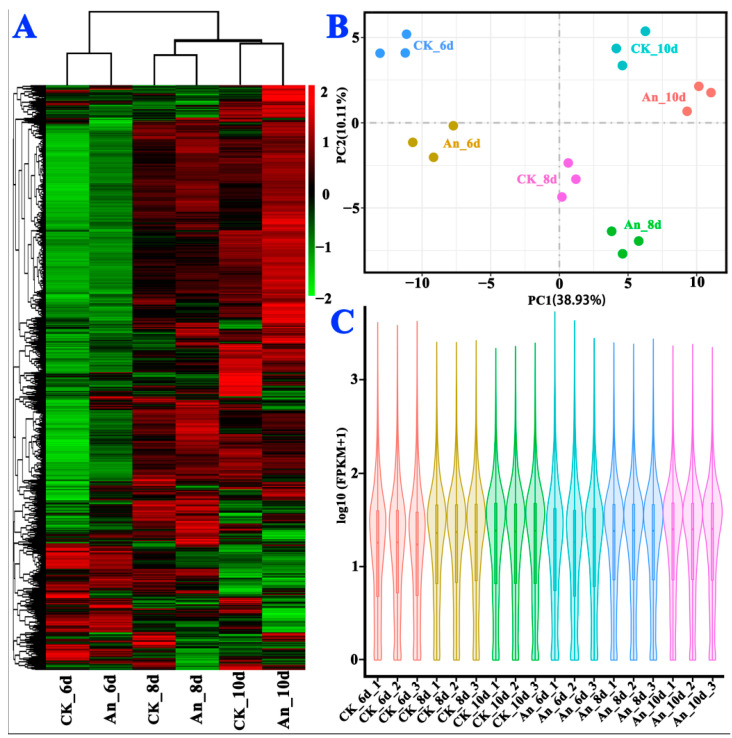
Cluster (**A**), principal component (**B**), and FPKM distribution (**C**) of DEGs in the RNA-seq samples.

Based on the expression level, which was reflected by fragments per kilobase of exon model per million (FPKM; Supplementary Table S2), cluster analysis, principal component analysis, and gene expression statistical analysis were performed (Figure 4). Three replicates in each group had close values (Figure 4B), and the distributions of the maximum, median, and minimum expression values of all genes were consistent among the samples (Figure 4C), indicating that differences among the replicate samples of each group were negligible. In other words, all the biological replicates presented excellent repeatability.

In addition, all the samples had no overlaps, and different groups were far apart (Figure 4B). The gene expression levels in different samples showed distinct dispersion (Figure 4C), indicating that cineole exerted a substantial effect on the gene expression of *A*. *cinnamomea*. However, the up-regulation and down-regulation of the DEGs are correlated with fermentation time (Figure 4A). In summary, the sampling time points for RNA-seq were reasonable, and each group showed good repeatability.

#### 2.4.3. Enrichment Analysis of DEGs

**Figure 5:**
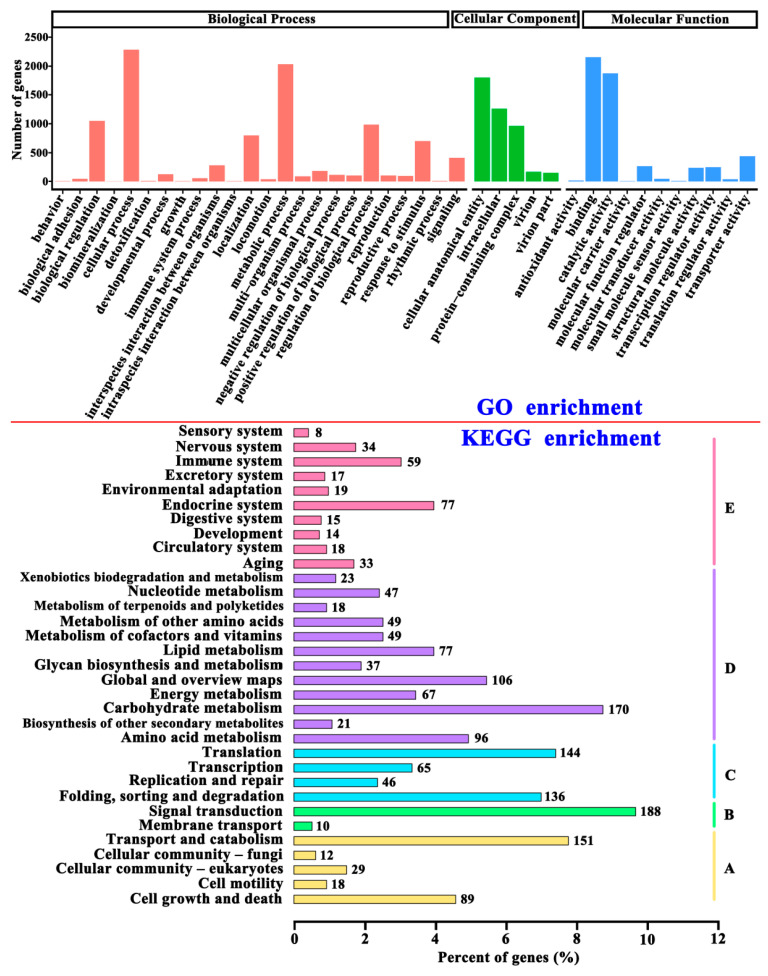
GO and KEGG classification enrichment analysis of the differential expression genes. For KEGG enrichment: A, cellular process; B, environmental information processing; C, genetic information processing; D, metabolism; E, organismal system.

Enrichment analysis results (Figure 5) showed that the DEGs were involved in transporter activity, molecular carrier activity, cell wall/membrane/envelope biogenesis, intracellular trafficking, secretion, vesicular transport, signal transduction mechanisms, environmental information processing, genetic information processing, transcription, and translation. A total of 188 DEGs were involved in signal transduction. Some signaling pathways, such as cell wall integrity (CWI) signaling pathways, affect the growth, reproduction, and sporulation of filamentous fungi [16]. Moreover, it was reported that cineole promotes fungal sporulation [17] and may affect the composition of membrane lipids by changing the expression of genes related to membrane components [18]. The available evidence supports the idea that cineole may change the membrane functional state of *A*. *cinnamomea* and then play a role in promoting its sporulation.

### 2.5. Bioinformatic Analysis

According to relevant references [16,18,19,20,21,22,23,24,25,26,27,28,29,30,31,32,33,34,35] and the unigene database (Supplementary Table S3) of RNA-seq, 25 genes (Table 2) may be involved in the process by which cineole promotes the sporulation of *A*. *cinnamomea*: *velB*, *flbA*, *pkaA*, *flbD*, *wetA*, *abaA*, *stuA*, *brlA*, *slt2*, *areA*, *pmk1*, *hog1*, *wsc1*, *Mid2*, *bck1*, *mkk1*, *pkc1*, *rlm1*, *dgk1*, *cki1*, *mep1*, *cdc37*, *PSD*, *hsp90*, and *ncp1*. Among them, *flbA*, *flbD*, *brlA*, *abaA*, *wetA*, *velB*, *stuA*, *areA*, and *pkaA* are the genes involved in the FluG-mediated asexual sporulation signaling pathway of *A*. *cinnamomea* [5].

The mitogen-activated protein kinase (MAPK) pathway plays an irreplaceable role in fungal growth and development [19,20]. Slt2-MAPK, Hog1-MAPK, and Fus3/Kss1-MAPK are the three MAPK pathways discovered in filamentous fungi. The Slt2-MAPK signaling pathway, also known as the CWI signaling pathway, is involved in the growth, reproduction, signal transmission, and sporulation of filamentous fungi [16,21], and Wsc1 and Mid2 are the cellular sensors of the CWI pathway [22]. Futagami et al. [23] found that WscA and WscB, which are the homologous proteins of Wsc1, play an important role in the CWI signaling pathway and regulation of conidium formation in *Aspergillus nidulans*. The deletion of *wscA* and *wscB* remarkably inhibited the growth and sporulation of *A*. *nidulans*. In addition, Mid2 is involved in the CWI pathway in *A*. *nidulans* [24]. In the local RNA-seq database, the genes of *Wsc1* and *Mid2* were successfully matched to Cluster-196.3943 and Cluster-196.2321, respectively (Table 2).

Xie et al. [25,26] found that the deletion of *bck1*, *mkk1*, and *slt2* in the CWI pathway markedly affected the mycelial growth, development, and pathogenicity of *Arthrobotrys oligospora*. The ΔAoSlt2, ΔAoBck1, and ΔAoMkk1 mutant strains completely lost their ability to produce spores. In *Beauveria bassiana*, *bck1*, *mkk1*, and *slt2* maintain the integrity of the cell wall and positively regulate growth and sporulation [27]. Protein kinase C (PKC) is essential for the growth and development of *Magnaporthe oryzae* and plays a key role in spore germination, cell wall biogenesis, growth, and mycelial development. The ability of *Pkc1*^s^ (*Pkc1* gene silencing) mutant strains to produce spores sharply decreases in *M*. *oryzae* [28]. Tan et al. [29] found that *rlm1* is involved in maintaining the cell wall integrity of *Aspergillus flavus* and affects spore production. The deletion of *rlm1* considerably reduces the transcription levels of sporulation-related genes. In the local RNA-seq database, *bck1*, *mkk1*, *slt2*, *Pkc1*, and *rlm1* were successfully matched to Cluster-196.3873, Cluster-196.4632, Cluster-196.3585, Cluster-196.1593, and Cluster-196.4376, respectively (Table 2).

Cineole may affect the composition of membrane lipids by changing the expression levels of genes related to cell membrane components. Cineole treatment inhibits the formation of the biofilm of the *Fusarium solani* species complex (*F. solani*) and alters the functional state of the cell membrane; moreover, the expression of phosphatidylserine decarboxylase (PSD) is down-regulated, and the expression levels of diacylglycerol kinase (DGK1) and choline kinase (CKI1) are up-regulated [18]. In the local RNA-seq database, *PSD*, *DGK1*, and *CKI1* were successfully matched to Cluster-196.940, Cluster-196.4865, and Cluster-196.4571, respectively (Table 2). Heat shock protein 90 (Hsp90) is one of the signature proteins of cells responding to heat stress. Bui et al. [30] found that Hsp90 plays a crucial role in the growth, reproduction, and virulence of *Fusarium graminearum*. FgHsp90 has a high expression level during conidium formation and promotes sporulation by up-regulating sporulation-related genes (stuA, *abaA,* and *wetA*) [30].

Furthermore, FgHsp90 accumulates in the late stage of sporulation, indicating that it is involved in spore formation as a transcriptional regulator. The Hsp90 co-chaperone Cdc37 interacts with Hog1 and Slt2 and controls the CWI pathway and cascade functions of HOG in *S*. *cerevisiae* [31]. Zhang et al. [32] found that the deletion of *hog1* and *slt2* resulted in a significant decrease in the sporulation rate of *Alternaria alternata*. The *pmk1* gene in the Fus3/Kss1-MAPK pathway affects the growth, sporulation, and pathogenesis of *Colletotrichum truncatum*, and the deletion of *pmk1* results in a sharp decrease in sporulation rate [33]. In the local RNA-seq database, *hsp90*, *cdc37*, *hog1*, and *pmk1* were successfully matched to Cluster-196.2224, Cluster-196.4302, Cluster-196.3144, and Cluster-196.3970, respectively (Table 2).

The GATA transcription factor AreA plays an important role in the growth, development, nitrogen metabolism, and pathogenicity of *F*. *graminearum*. The deletion of *areA* resulted in a marked reduction in the sporulation rate of *F*. *graminearum* and significantly reduced the expression of *mep* (an ammonium permease) and the activity of PKA [34]. AreA is the key regulator of nitrogen metabolism, regulates the expression of *mep*, and affects the MAPK and PKA pathways [34]. NCP1 is a transcription factor with a C_2_H_2_ zinc finger structure, and the deletion of NCP1 causes a significant reduction in the sporulation rate of *Metarhizium acridum* [35]. In the local RNA-seq database, *areA*, *mep*, and *NCP1* were successfully matched to Cluster-196.4221, Cluster-196.4962, and Clus ter-196.397, respectively (Table 2).

### 2.6. RT-qPCR Analysis

**Figure 6:**
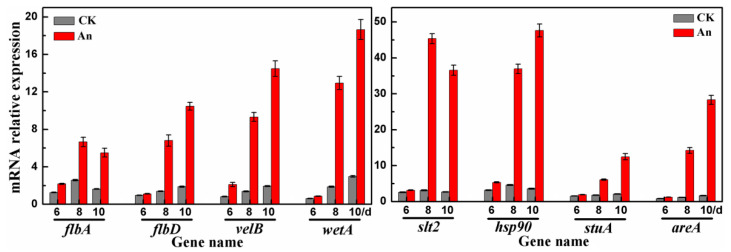
Expression levels of relevant genes in different samples.

The expression levels (FPKM values) of the genes listed in Table 2 in different samples were compared. *flbA*, *flbD*, *velB*, and *wetA* in the FluG-mediated sporulation signaling pathway and *slt2*, *hsp90*, *stuA*, and *areA* underwent remarkably different changes after cineole treatment. Thus, RT-qPCR analysis was used to further measure and verify the expression levels of the genes in each sample. Their expression levels increased 3–17 times after cineole treatment (Figure 6). The expression levels of *velB* increased 7.5 times; *wetA*, 7 times; *slt2* and *hsp90*, 14 times; and *areA*, 17 times. The RT-qPCR results showed that the fold changes in the expression levels of the related genes were even greater than the fold changes in the FPKM values from the RNA-seq database (Supplementary Table S2). The fact that the expression levels dramatically increased after cineole treatment supports the hypothesis that *flbA*, *flbD*, *velB*, *wetA*, *slt2*, *hsp90*, *stuA*, and *areA* may play a key regulatory role in the cineole-induced sporulation of *A*. *cinnamomea*.

### 2.7. Putative Signaling Pathway of the Cineole-Promoted Sporulation of A. cinnamomea

The CWI signaling pathway is important for the growth, reproduction, signal transmission, and pathogenicity of fungi. The cell sensors Wsc1 and Mid2 are located upstream of the CWI pathway and receive external signals [22]. Extracellular signals are then transmitted through the CWI pathway and promote the transcription of genes related to CWI [22]. Pkc1 is a serine or threonine protein kinase upstream of the CWI pathway and plays a key role [36]. The sporulation of a Pkc1 mutant strain is significantly reduced in *M. oryzae*, indicating that Pkc1 plays an important role in sporulation [28]. The *rlm1* gene is located downstream of the CWI pathway. The deletion of *rlm1* causes a marked decrease in the sporulation rate of *A. flavus* [29]. Rlm1 is a MADS box transcription factor [37] and is similar to the transcription factor AbaA. In the central developmental pathway of the FluG-mediated sporulation pathway in *A*. *cinnamomea*, *abaA* directly acts on *wetA* and promotes its expression [5]. In the present study, the expression of *wetA* sharply increased in the samples treated with cineole (Figure 6). Therefore, it was supposed that *rlm1* has the same function as *abaA* and directly promotes the expression of *wetA*.

The available evidence supports the hypothesis that when cineole was present in the external environment, Wsc1 and Mid2 sensors on the cell membrane of *A*. *cinnamomea* received the signal and transmitted it to Pkc1. Then, the signal promoted the expression of related genes in the CWI pathway through a series of cascade reactions and induced cell wall synthesis and spore maturation in *A*. *cinnamomea*. The activation of the CWI pathway promotes the expression of the downstream gene *rlm1*, which further promotes the up-regulation of *wetA* and ultimately promotes the sporulation of *A*. *cinnamomea*. In addition, *velB* and *wetA* promote each other’s expression [5], thus promoting cell wall synthesis and spore maturation in *A*. *cinnamomea*. In this study, the expression levels of *velB* and *wetA* in samples treated with cineole were sharply up-regulated compared with the control group. This fact suggests that *velB* and *wetA* play an important role in the cineole-promoted sporulation of *A*. *cinnamomea*.

The CWI signaling pathway is activated not only by stress on the cell wall but also by many other conditions, such as heat stress, hypotonic shock, oxidative stress, and high or low pH, indicating that CWI is involved in some stress responses and cellular processes that have no direct relationship with the cell wall [38]. The heat shock activation of the CWI pathway depends on the sensors of Wsc1 and Mid2 [39,40]. In *Saccharomyces cerevisiae*, plasma membrane stretching caused by an increase in cell membrane functional state and the accumulation of intracellular permeates is considered the mechanism by which CWI activates a response to heat stress [39,41]. Cineole may affect the cell membrane functional state of *F. solani* by changing the expression of membrane component-related genes, such as PSD, DGK1, and CKI1 [18]. In *S. cerevisiae*, the interaction of Slt2 with Cdc37 and Hsp90 is critical for Slt2-dependent downstream responses, such as the activation of transcription factor *rlm1* [30].

In this study, the expression levels of *hsp90* and *slt2* were sharply up-regulated in the samples treated with cineole (Figure 6). The available evidence supports the hypothesis that cineole changed the permeability of the *A*. *cinnamomea* cell membrane and activated the expression of *hsp90* and *cdc37*, thereby promoting the Slt2-CWI signaling pathway, activating *rlm1*, and finally promoting sporulation of *A*. *cinnamomea*. In addition, Hsp90 was massively expressed during the sporulation of *F. graminearum* and participated in the formation of spores by up-regulating the expression of *stuA* [30]. In the present study, the expression of *stuA* markedly increased in the samples treated with cineole (Figure 6). Therefore, it was supposed that the expression of *stuA* is promoted by Hsp90 co-chaperone Cdc37 and further promotes the expression of *brlA* [5], ultimately promoting the sporulation of *A*. *cinnamomea*.

NCP1, a C_2_H_2_ zinc finger structural transcription factor, affects the sporulation of filamentous fungi. In *M. acridum*, the deletion of *MaNCP1* decreases its ability to utilize nitrate, ammonium, and glutamine and reduces the expression levels of genes related to nitrate assimilation, indicating that MaNCP1 is involved in the regulation of nitrogen utilization [35]. The sporulation capacity of the ∆*MaNCP1* mutant strain was significantly reduced. Further studies have indicated that MaAreA is a key regulator of the nitrogen metabolism pathway and a downstream target gene of MaNCP1 [35]. In *F. graminearum*, the deletion of *12rea* not only dramatically inhibits its growth, sporulation, and spore germination but also significantly reduces the expression of *mep* and the activity of PKA [34]. AreA affects the PKA pathway by regulating the expression of MEP in *F. graminearum* [34]. In this study, the expression levels of *12rea*, *flbA*, and *flbD* were markedly up-regulated in mycelium samples treated with cineole (Figure 6). The available evidence supports the hypothesis that the C_2_H_2_ zinc finger transcription factor of NCP1 promotes the expression of its downstream target gene *areA* and then promotes the expression of *flbA* and *flbD* by inducing the expression of MEP. Meanwhile, MEP inhibits the expression of *pkaA*, and the up-regulation of *flbD* promotes the sporulation of *A. cinnamomea.* The up-regulation of *flbA* and down-regulation of *pkaA* alleviate the inhibition of the sporulation pathway and indirectly promote the sporulation of *A. cinnamomea*.

**Figure 7:**
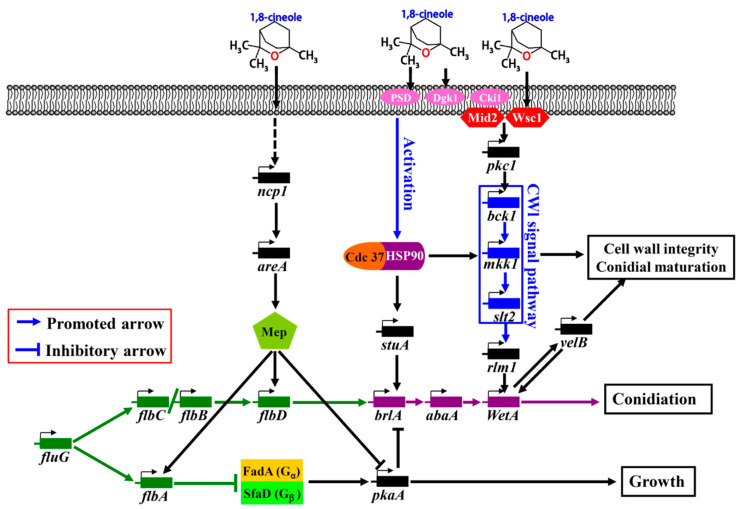
The putative signal pathway of the 1,8-cineole- and FluG-mediated sporulation of *A*. *cinnamomea* in submerged fermentation.

In summary, based on comparative transcriptomic analyses and academic publications, the signaling pathway of the cineole-promoted sporulation of *A*. *cinnamomea* was proposed as follows (Figure 7): The cell receptors of Wsc1 and Mid2 receive the signal of cineole and send it to the CWI signal pathway through Pkc1; the CWI signal pathway then promotes the expression of *rlm1* and *wetA* through cascade reactions and finally promotes the sporulation of AcSmF. *velB* and *wetA* promote each other’s expression and then promote the cell wall synthesis and spore maturation of *A. cinnamomea*. Cineole increases the membrane functional state of *A. cinnamomea*, and heat stress is activated by the CWI signal pathway. Subsequently, Hsp90 cooperates with its chaperone, Cdc37, to promote the expression of *stuA* and the relevant genes in the CWI pathway. *stuA* further promotes the expression of *brlA* and the sporulation of AcSmF. In addition, cineole acts indirectly on NCP1 and promotes its expression, further promoting the expression of *areA*. AreA promotes the expression of *flbA* and *flbD* through the ammonium permease of MEP and inhibits the expression of *pkaA*. The up-regulated expression of *flbD* promotes the sporulation of AcSmF, whereas the up-regulated expression of *flbA* and the down-regulated expression of *pkaA* indirectly promote the sporulation of AcSmF by inhibiting the PKA pathway.

## 3. Materials and Methods

### 3.1. Materials

The *A*. *cinnamomea* strain (ATCC 200183) was purchased from the American Type Culture Collection (Manassas, VA, USA), and the wild wood of *C. kanehirae* Hay was purchased from Fujian HaoTian Biological Technology Co., Ltd. (Fuzhou, China).

### 3.2. Submerged Fermentation of A. cinnamomea

*A*. *cinnamomea* was cultured in accordance with a previously described method [5]. The spore concentration of a spore suspension (i.e., inoculum) was calculated with a hemocytometer and optical microscope. Then, an appropriate amount of inoculum was added to a 500 mL Erlenmeyer flask containing 100 mL of a medium (20.0 g/L glucose, 4.0 g/L yeast extract powder, 3.0 g/L KH_2_PO_4_, and 1.5 g/L MgSO_4_; initial pH 4.5). The concentration of the spores in the inoculum was 1.0 × 10^6^ spores/mL. Finally, *A*. *cinnamomea* was fermented at 26 °C and 150 r/min for 10–12 days.

### 3.3. Effects of Different Additives on the Sporulation and Biomass of AcSmF

#### 3.3.1. Preparation of *C. kanehirae* Extracts

*C*. *kanehirae* wood was shredded into sawdust. Then, 20 g of sawdust were soaked in 400 mL of methanol, ethyl acetate, chloroform, or petroleum ether, and the mixtures were shaken at 300 r/min for 3 h. The supernatants were collected through centrifugation (6000 r/min, 10 min, 4 °C) and dried at 75 °C. Finally, the methanol extract of *C*. *kanehirae* (CkME), ethyl acetate extract of *C. kanehirae* (CkEE), chloroform extract of *C. kanehirae* (CkCE), and petroleum ether extract of *C. kanehirae* (CkPE) were obtained.

#### 3.3.2. Effects of Additives on the Sporulation and Biomass of AcSmF

*C*. *kanehirae* extracts were dissolved in dimethyl sulfoxide (DMSO) to prepare a solution with a concentration of 50 mg/mL; cineole was dissolved in DMSO to a concentration of 1 mg/mL; and L-histidinol or deoxyuridine was dissolved in deionized water to a concentration of 5 mg/mL. All the solutions were filtered and sterilized with a sterile filter membrane with a diameter of 0.45 µm. When *A*. *cinnamomea* was fermented for 5 days, an appropriate volume of CkME, CkEE, CkCE, CkPE, cineole, L-histidinol, or deoxyuridine solution was added to the medium. Then, each flask was sampled regularly every day to detect the sporulation from the 6th day. The biomass was detected on the 10th day. The same volumes of DMSO or sterile water (blank control) were added to the mediums.

### 3.4. LC-MS/MS Analysis for CkCE

The concentration of CkCE was adjusted to 20 mg/L, and the extract was filtered with a membrane 0.22 μm in diameter. Then, an appropriate amount of CkCE solution was added to the sample vial for LC-MS/MS analysis at Suzhou Panomix Biomedical Tech Co., Ltd. (Suzhou, China). The following procedures [42] were performed: Liquid chromatography analysis was performed on a Vanquish UHPLC System (ThermoFisher Scientific, Waltham, MA, USA). Chromatography was carried out with an ACQUITY UPLC HSS T3 (150 mm × 2.1 mm, 1.8 μm; Waters, Milford, MA, USA). The column was maintained at 40 °C, and the flow rate and injection volume were set at 0.25 mL/min and 2 μL, respectively. The mass spectrometric detection of metabolites was performed with Q Exactive HF-X (ThermoFisher Scientific, USA) with an ESI ion source. Simultaneous MS1 and MS/MS (Full MS-ddMS2 mode, data-dependent MS/MS) acquisition was performed with the following parameters: sheath gas pressure, 30 arb; auxiliary gas flow, 10 arb; spray voltage, 3.50 and −2.50 kV for ESI(+) and ESI(−), respectively; capillary temperature, 325 °C; MS1 range, m/z positive 81–1000, negative 91–1000; MS1 resolving power, 60,000 FWHM; number of data-dependent scans per cycle, 8; MS/MS resolving power, 15,000 FWHM; normalized collision energy, 30 eV; dynamic exclusion time, automatic.

### 3.5. Sample Preparation for RNA-Seq

*A*. *cinnamomea* mycelia for RNA-seq were prepared in accordance with a previously described method [5]. *A*. *cinnamomea* mycelium pellets cultured in the presence or absence of cineole for 6, 8, and 10 days were collected through filtering with four layers of gauze. Then, the mycelium pellets were washed with an ethylene diamine tetraacetic acid buffer solution (pH 8.0) and snap frozen with liquid nitrogen. Three biological replicates were collected at each time point from each group.

### 3.6. RNA-Seq and Bioinformatic Analysis

High-throughput sequencing of the *A*. *cinnamomea* samples was performed on the Illumina NovaSeq 6000 system (San Diego, CA, USA). The procedure was performed at Beijing Novogene Biotechnology Co., Ltd. (Beijing, China). The raw data of all 18 samples were deposited in the Sequence Read Archive with the accession number PRJNA1031605 (NCBI). The quality of the reads was controlled with FASTP software (version 0.19.7) and assembled with Trinity software (version 2.6.6) [43]. The unigene data (Supplementary Table S1) of *A*. *cinnamomea* were matched with the genome data (Accession number: GCA_000766995.1; NCBI) of *A*. *cinnamomea*. The differentially expressed genes (DEGs) were obtained with the H-Cluster algorithm by Corset (https://code.google.com/p/corset-project accessed on 16 February 2023) [44]. The NCBI, GO, and KEGG databases were used in annotating the DEGs and determining their functions.

### 3.7. RT-qPCR Analysis

RT-qPCR analysis was performed in accordance with previously described methods and conditions [5]. The 2^−ΔΔCt^ calculation method [45] was used in quantifying the transcription levels of genes, and the 18S rRNA sequence of *A. cinnamomea* was used as the internal reference. The RT-qPCR primer sequences of genes are listed in Supplementary Table S4.

### 3.8. Statistical Analysis of Data

Three replicates of each experimental group were prepared, and the data were presented as means ± SDs. A one-way ANOVA was used for significance analysis. SPASS PASW Statistics 18.0 was used, and *p* < 0.05 indicated a significant difference.

## 4. Conclusions

CkCE was found to significantly promote the sporulation of AcSmF. LC-MS/MS analysis and verification experiments uncovered that the main compound that promoted sporulation of *A*. *cinnamomea* in CkCE is 1,8-cineole. Subsequently, RNA-seq and RT-qPCR technologies were used to predict the molecular regulatory mechanism underlying the cineole-promoted sporulation of *A*. *cinnamomea*. Briefly, it was supposed that cineole promoted the expression of *wetA* through cell sensors (Wsc1 and Mid2), Pkc1, the CWI signaling pathway, and *rlm1*, thereby promoting spore maturation and sporulation. At the same time, cineole changed the functional state of the cell membrane and activated the heat stress response, which promoted sporulation of *A*. *cinnamome* through Hsp90, Cdc37, *stuA*, and *brlA*. In addition, cineole also promoted the expression of *flbA* and *flbD* and inhibited the expression of *pkaA*, further promoting the sporulation of *A*. *cinnamome*. However, due to the lack of a mature and stable genetic manipulation system, the functions of the relevant genes were not verified by deletion or overexpression. Nevertheless, the conclusion of the present study was obtained by using rich RNA-seq data, data from rigorous bioinformatics analysis, and abundant research publications. This study further supplements and improves understanding of the molecular regulation mechanism underlying sporulation for *A*. *cinnamome* and is useful in the regulation of AcSmF sporulation.

## Figures and Tables

**Table 1:** Top 20 compounds in the chloroform extract of *C. kanehirae*.

No.	Name	Formula	CAS NO.	Content
1	1,8-Cineole	C_10_H_18_O	470-82-6	20.49%
2	L-Histidinol	C_6_H_11_N_3_O	1596-64-1	9.95%
3	Deoxyuridine	C_9_H_12_N_2_O_5_	54-42-2	9.45%
4	Catechol	C_6_H_6_O_2_	120-80-9	8.84%
5	(-)-beta-Pinene	C_10_H_16_	18172-67-3	4.97%
6	2-Chloro-L-phenylalanine	C_9_H_10_C_1_NO_2_	54793-54-3	4.36%
7	Phenyl acetate	C_8_H_8_O_2_	122-79-2	3.36%
8	Hydroquinone	C_6_H_6_O_2_	123-31-9	2.34%
9	m-chlorophenylpiperazine	C_10_H_13_C_1_N_2_	6640-24-0	2.06%
10	Fomepizole	C_4_H_6_N_2_	7554-65-6	2.03%
11	D-Ribose	C_5_H_10_O_5_	50-69-1	1.46%
12	Thiabendazole	C_10_H_7_N_3_S	148-79-8	1.43%
13	Genipin	C_11_H_14_O_5_	6902-77-8	1.17%
14	Baicalein	C_15_H_10_O_5_	491-67-8	0.99%
15	1-Aminocyclopropanecarboxylic Acid	C_4_H_7_NO_2_	22059-21-8	0.89%
16	Trans-Cinnamate	C_9_H_8_O_2_	1754-627-7	0.82%
17	Dihydroxyfumaric acid hydrate	C_4_H_4_O_6_	19926-38-0	0.77%
18	Picolinic acid	C_6_H_5_NO_2_	98-98-6	0.70%
19	Sinapyl alcohol	C_11_H_14_O_4_	537-33-7	0.66%
20	Phytosphingosine	C_18_H_39_NO_3_	554-62-1	0.65%

**Table 2:** The genes that may be involved in 1,8-cineole-promoting the sporulation of AcSmF.

Unigene ID	Genome ID	Gene Name	Accession Number	E Value	Score
Cluster-196.3786	ACg006274	*velB*	B0CXQ2.1	1 × 10^−102^	359
Cluster-196.2321	ACg005708	*flbA*	P38093.1	3 × 10^−5^	47
Cluster-196.4288	ACg006986	*pkaA*	ETI83732.1	4 × 10^−28^	122
Cluster-196.2514	ACg008440	*flbD*	JAC66949.1	1 × 10^−13^	74
Cluster-196.908	ACg000535	*wetA*	XP_016271747.1	6 × 10^−5^	42
Cluster-196.1773	ACg008470	*abaA*	EAA66521.1	6 × 10^−13^	73
Cluster-196.3966	ACg004592	*stuA*	KKF92291.1	6 × 10^−11^	65
Cluster-196.3292	ACg003227	*brlA*	A0A0F0I5G4.1	3 × 10^−9^	60
Cluster-196.3585	ACg007355	*slt2*	XM_027756054.1	1 × 10^−162^	571
Cluster-196.4221	ACg003505	*areA*	CP021224.1	2 × 10^−120^	446
Cluster-196.3970	ACg006851	*pmk1*	PCH35243.1	2 × 10^−24^	106
Cluster-196.3144	ACg008555	*hog1*	OCH90560.1	5 × 10^−34^	138
Cluster-196.3943	ACg007303	*wsc1*	NC_007198.1	2 × 10^−9^	59
Cluster-196.2321	ACg005707	*mid2*	NP_013436.1	3 × 10^−4^	37
Cluster-196.3873	ACg008261	*bck1*	NP_012440.1	2 × 10^−4^	35
Cluster-196.4632	ACg006849	*mkk1*	NW_007930837.1	8 × 10^−52^	203
Cluster-196.1593	ACg002614	*pkc1*	NP_009445.2	3 × 10^−6^	44
Cluster-196.4376	ACg002705	*rlm1*	XP_001387522.2	2 × 10^−9^	54
Cluster-196.4865	ACg003577	*dgk1*	KAI0930476.1	1 × 10^−8^	56
Cluster-196.4571	ACg008481	*cki1*	XP_024338459.1	1 × 10^−35^	145
Cluster-196.4962	ACg007312	*mep1*	NP_011636.3	6 × 10^−16^	76
Cluster-196.4302	ACg002703	*cdc37*	NP_595752.1	4 × 10^−14^	70
Cluster-196.940	ACg008458	*psd*	KAI0635493.1	8 × 10^−50^	193
Cluster-196.2224	-	*hsp90*	KZT67759.1	2 × 10^−54^	210
Cluster-196.397	ACg003414	*ncp1*	EJP65478.1	1 × 10^−4^	33

Note: “Unigene ID” is the code of the unigene generated in the process of software assembly; “Genome ID” is the code corresponding to the gene matched with the unigene in the *A. cinnamomea* genome (ACg); “-” indicates an unmatched gene in the *A. cinnamomea* genome database; “Accession number” is the NCBI number of the protein matched with a unigene in the local protein database; “*E* value” and “Score” are used to describe the matching between a unigene and the corresponding protein in the local protein database. If the *E* value is low, then the score is high and the matching degree is high. It was considered a successful match when the score ≥ 30.

## Data Availability

Data is contained within the article or Supplementary Materials.

## Supplementary Materials

The following supporting information can be downloaded at: https://www.mdpi.com/article/10.3390/molecules28227511/s1. Figure S1: the curve of sporulation with time cultured in the presence or absence of cineole for *A*. *cinnamomea* in submerged fermentation; Table S1: substances identified by LC-MS from chloroform extract of Cinnamomum kanehirae Hay; Table S2: the FPKM values of the unigenes and DEGs from RNA-seq; Table S3: the unigene database of Antrodia cinnamome by RNA-seq; Table S4: the primers used for RT-qPCR.
