# Supplementary material for: Comparative Transcriptomic Analyses Propose the Molecular Regulatory Mechanisms Underlying 1,8-Cineole from Cinnamomum kanehirae Hay and Promote the Asexual Sporulation of Antrodia cinnamomea in Submerged Fermentation
Source: Molecules. 2023 Nov 9;28(22):7511. doi: 10.3390/molecules28227511 (PMC10672923; doi:10.3390/molecules28227511)
Supplement: Supplementary file 1 [file molecules-28-07511-s001.zip › Figre S1 The curve of sporulation with time cultured in the presence or absence of cineole for A. cinnamomea in submerged fermentation.pdf]

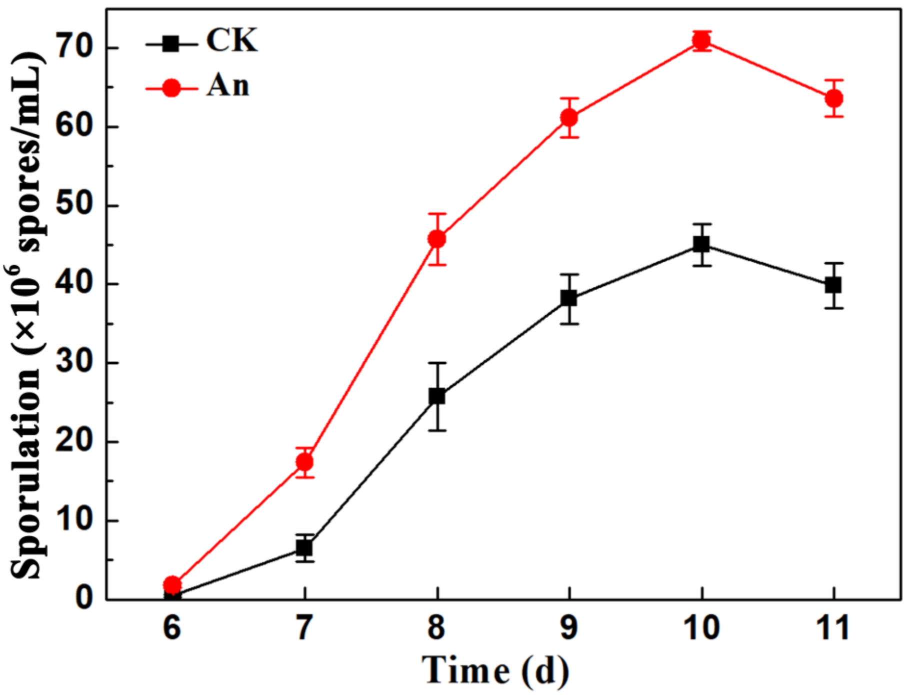

Figure S1. The curve of sporulation with time cultured in the presence or absence of cineole for *A. cinnamomea* in submerged fermentation

Note: “CK” means blank control; “An” means cultured with 500 μg/L of 1,8-Cineole.
