# Supplementary material for: Comparative Transcriptomic Analyses Propose the Molecular Regulatory Mechanisms Underlying 1,8-Cineole from Cinnamomum kanehirae Hay and Promote the Asexual Sporulation of Antrodia cinnamomea in Submerged Fermentation
Source: Molecules. 2023 Nov 9;28(22):7511. doi: 10.3390/molecules28227511 (PMC10672923; doi:10.3390/molecules28227511)
Supplement: Supplementary file 1 [file molecules-28-07511-s001.zip › Table S4 The Primers used for RT-qPCR.pdf]

Table S4. The Primers used for RT-qPCR

Gene name	Upstream Primer (5'→3')	Downstream Primer (5'→3')	Product (bp)
flbA	TGGCGACTGACTCTGTTCCC	CACCGATGGCGTAGATGTTCC	134
flbD	AATGTCTGAAGGTCGTGATGCC	GCCGTATCGTTAGCCGTATGG	126
velB	AATCGTTCTGGTCGGCATAGC	GGATAGACTTGAGCAGGATAGGC	128
wetA	TTTCCTGTTCCGTCCGTAAGC	GTGGTTGAGGTAGGGATTGATGG	112
stuA	GGTTGAATGCGACACAGATTTTG	TGATATTTCCCATAACCACCTTGC	123
hsp90	ACTGAGGAGTATGCTGCTTTCTAC	CGCTTAGGAACGAAGAGGATGG	122
areA	GTAGAGTGAGGCAAGGCAGATG	TGTCCAATTCAGTCCGCATACC	139
slt2	ATCTCCTTTAGAAGACATC	ATCTCCTTTAGAAGACATC	103
18S rRNA	GCTGGTCGCTGGCTTCTTAG	CGCTGGCTCTGTCAGTGTAG	123
